# Supplementary material for: Aeromonas in South Asia: genomic insights into an environmental pathogen and reservoir of antimicrobial resistance
Source: Nat Commun. 2026 Jan 31;17:2214. doi: 10.1038/s41467-026-68712-w (PMC12963386; doi:10.1038/s41467-026-68712-w)
Supplement: Supplementary file 1 — Supplementary Information [file 41467_2026_68712_MOESM1_ESM.pdf]

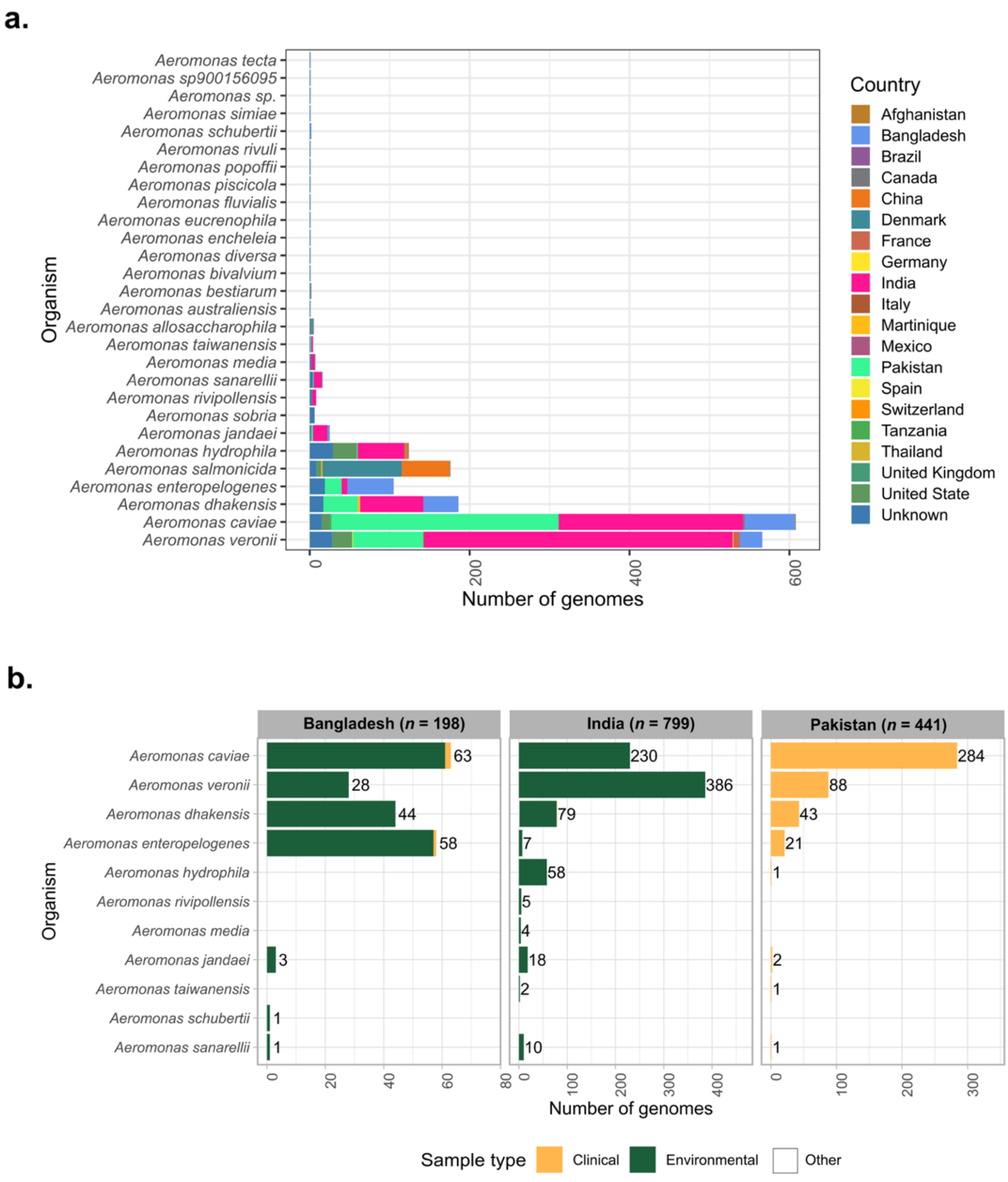

Supplementary Fig. 1: **Geographic distribution of *Aeromonas* species genomes included in this study.** (a) A total of 1,853 *Aeromonas* species genomes, including 996 sequenced in this study. Coloured bars represent various *Aeromonas* species across different countries (see key). (b) A subset of 1,438 *Aeromonas* species genomes distributed across South Asia, including Bangladesh, India, and Pakistan. The number in front of each bar indicates the number of genomes for the respective species in each country, and the bars are coloured by sample type (see key).

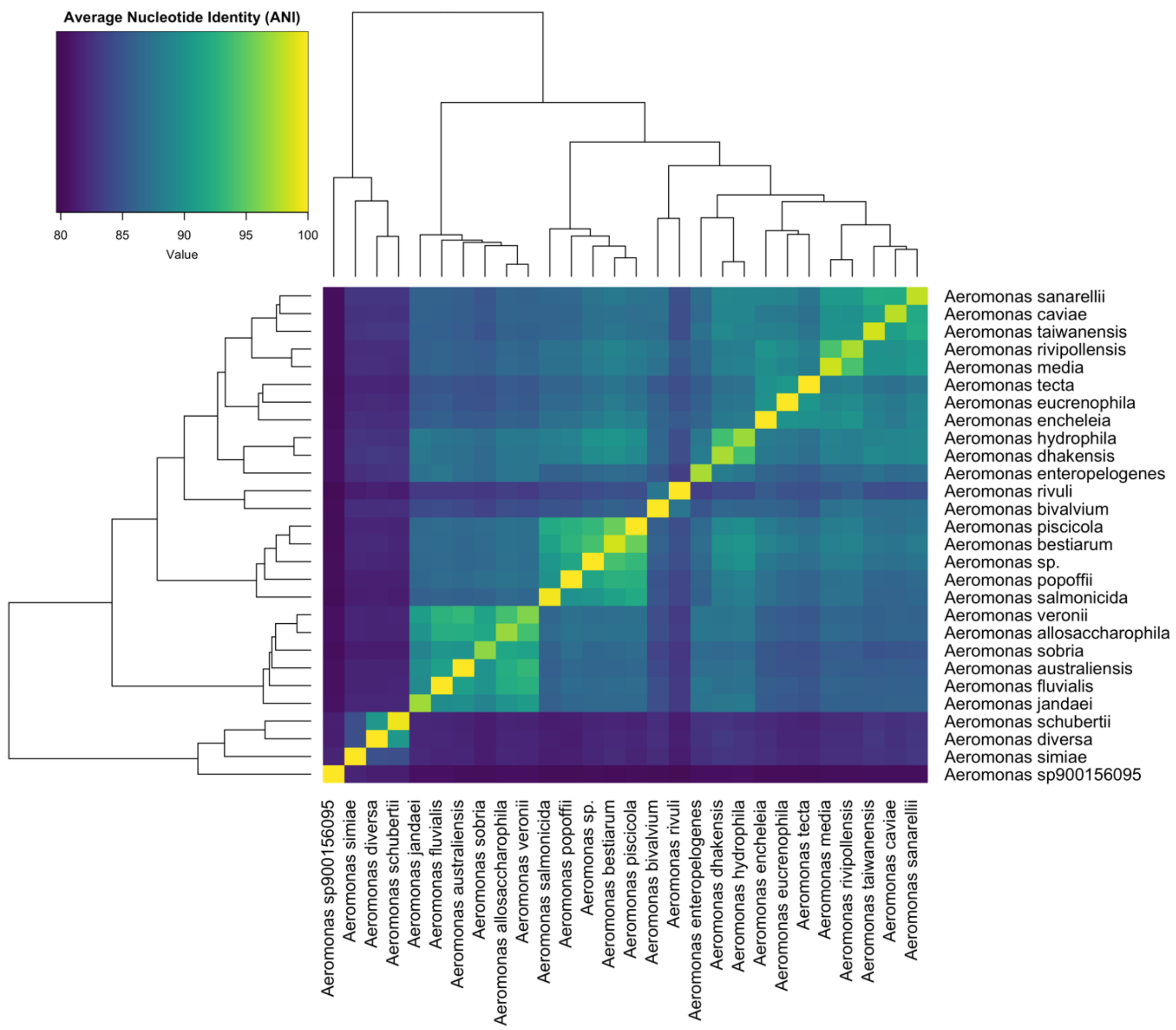

Supplementary Fig. 2: The Average Nucleotide Identity (ANI) among 1,853 *Aeromonas* spp. genomes. The colour-coded heatmap illustrates the pairwise genomic relatedness among the genomes, based on the average ANI values. The vertical dendrogram on the sides demonstrates the hierarchical clustering of genomes according to genetic similarities. The colour scale bar indicates FastANI scores, with higher scores indicating greater similarity.

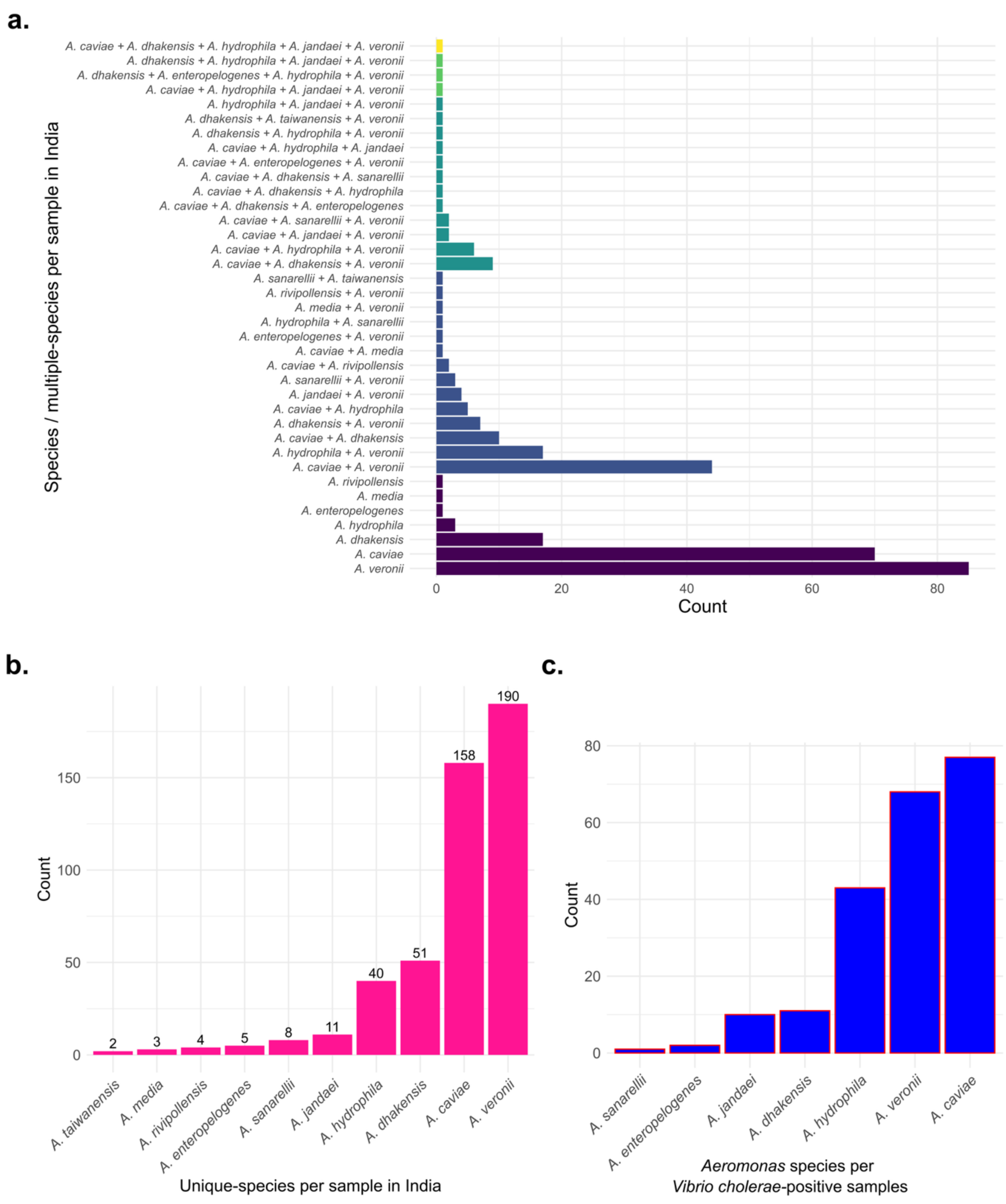

Supplementary Fig. 3: *Aeromonas* species genomes sequenced from positive water samples in Northern India. (a) Distribution of *Aeromonas* species (*y*-axis) based on the presence of single or multiple species per sample (*x*-axis). Each bar represents a combination of species within a sample. (b) Abundance of *Aeromonas* species across Northern India, with one representative genome per species per sample included to account for multiple colonies sequenced per sample. Species are shown on the *x*-axis and their overall counts on the *y*-axis. (c) Co-occurrence of *Aeromonas* species in 91 *Vibrio cholerae*–positive samples, with species on the *x*-axis and their counts on the *y*-axis.

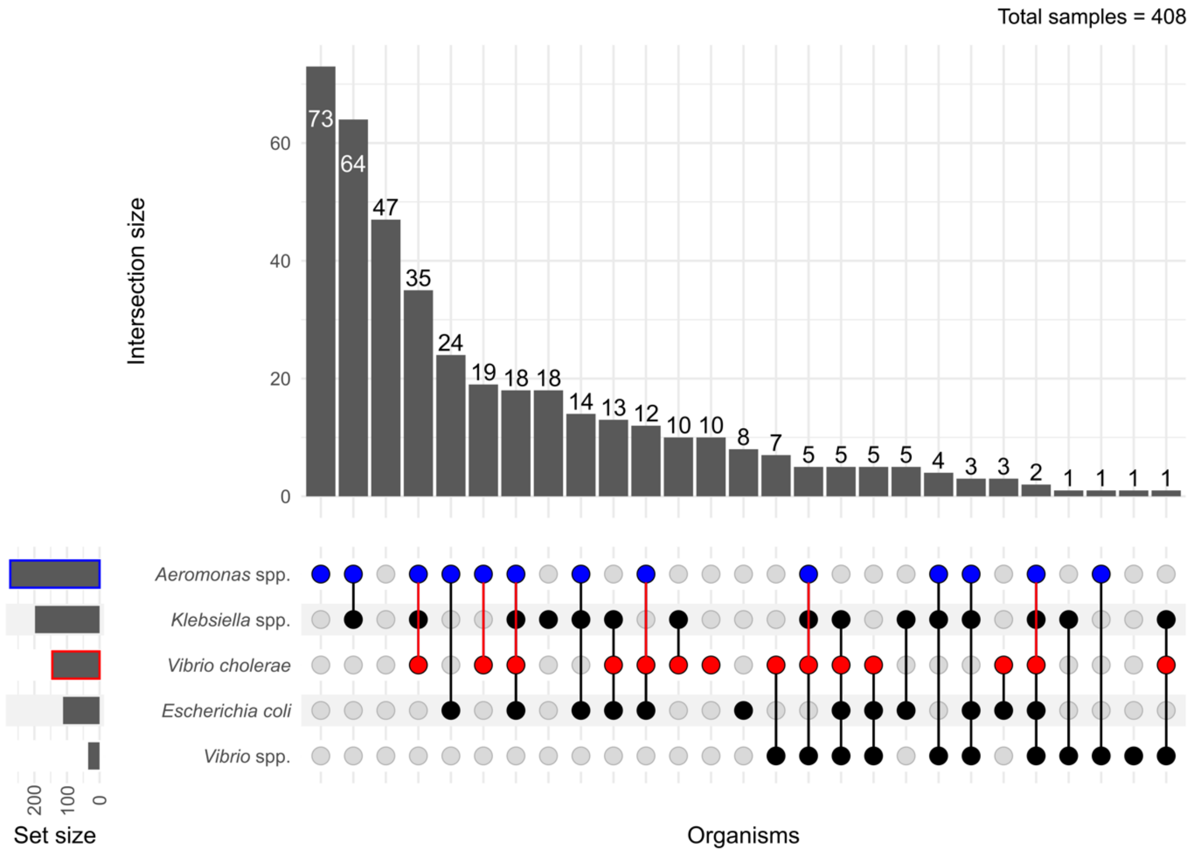

Supplementary Fig. 4: Co-occurrence of different bacteria in 408 water samples from Northern India as determined by culturing them on selective media for *V. cholerae* and enteric pathogens (see method).

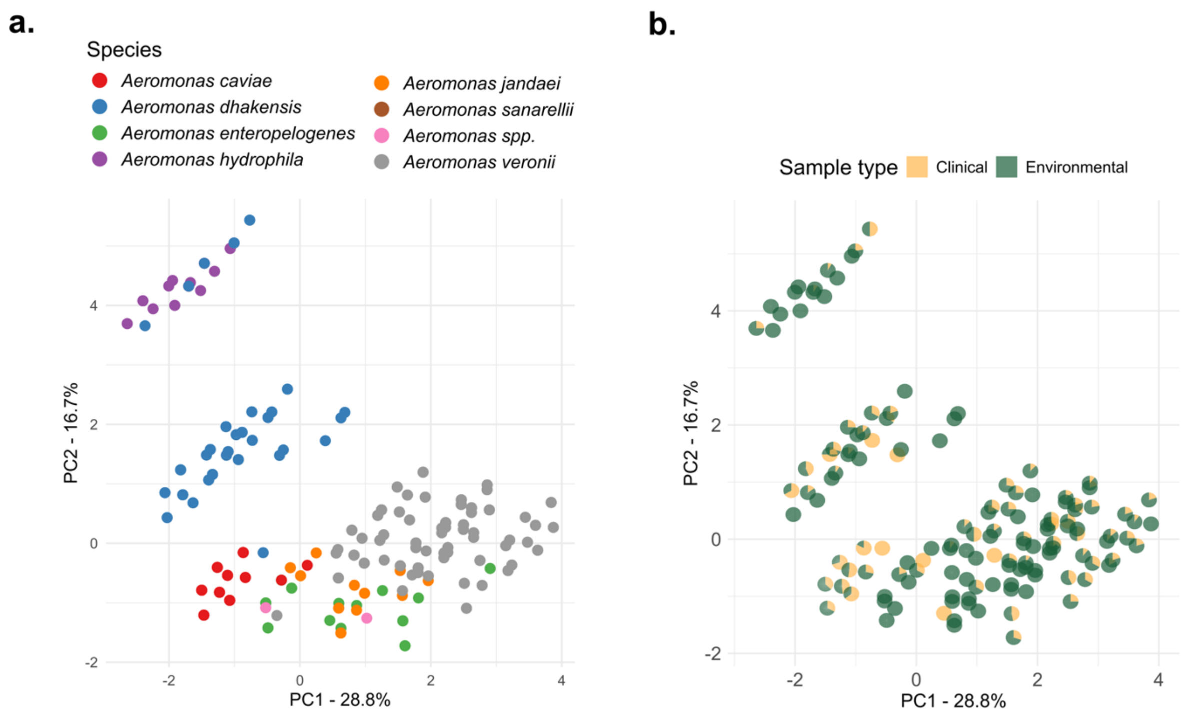

Supplementary Fig. 5: Distribution of virulence genes across 1,438 *Aeromonas* species isolates from South Asia, including Bangladesh, India, and Pakistan. (a) Principal Component Analysis (PCA) of virulence genes across different *Aeromonas* species (see key for coloured dots representing species). (b) Principal Component Analysis (PCA) of virulence genes across clinical and environmental isolates (see key for coloured dots representing sample type).

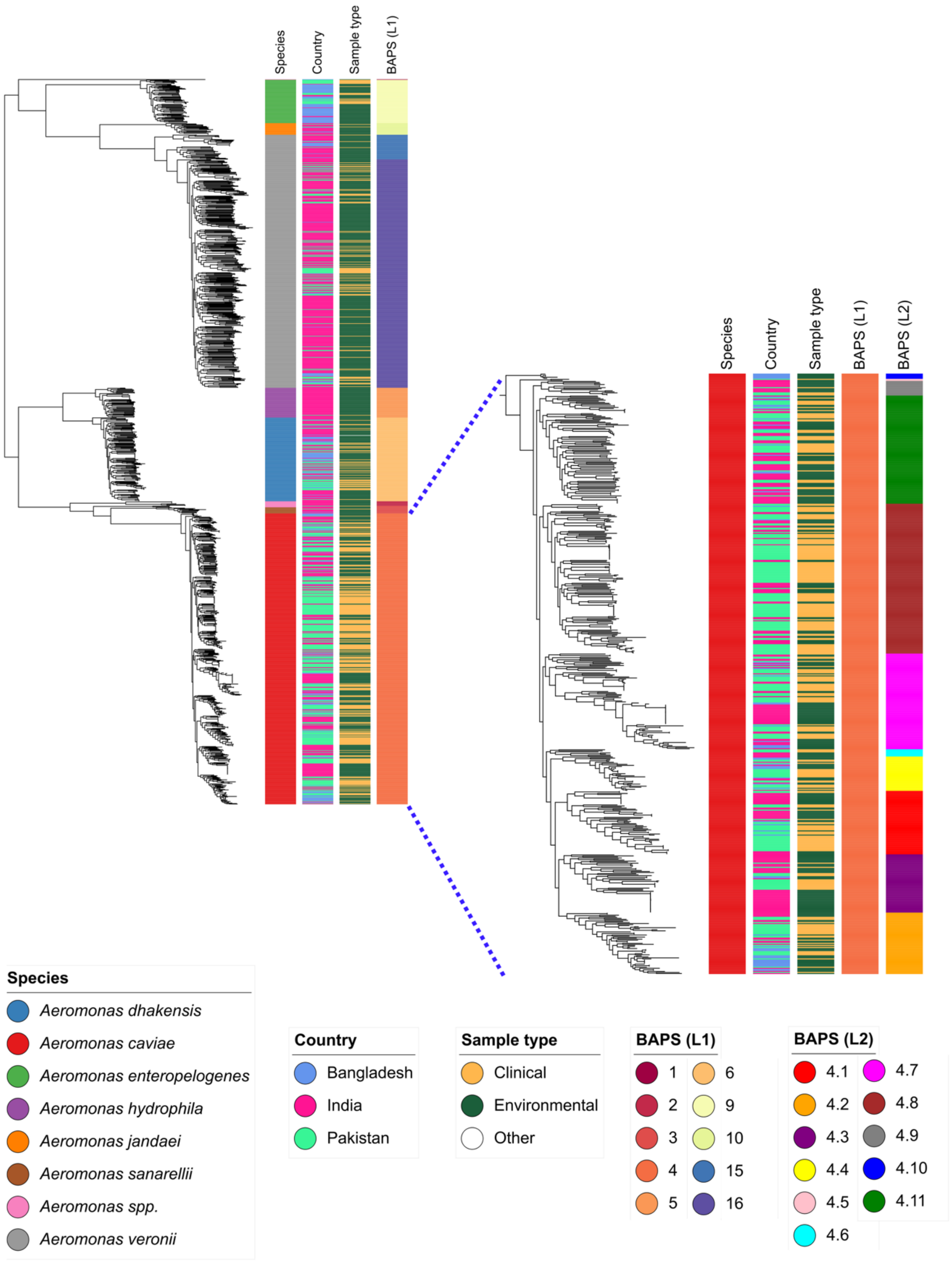

Supplementary Fig. 6: A maximum likelihood phylogenetic tree of 1,438 *Aeromonas* species genomes from South Asia (Bangladesh, India, and Pakistan), based on 2,067 core genes. A subtree containing only *A. caviae* is highlighted separately with a dotted line. Refer to the key for interpretation of the colour strips alongside. The scale bars indicate an evolutionary distance of 0.1 nucleotide substitutions per site for the whole tree and 0.01 substitutions per site for the *A. caviae* subtree.

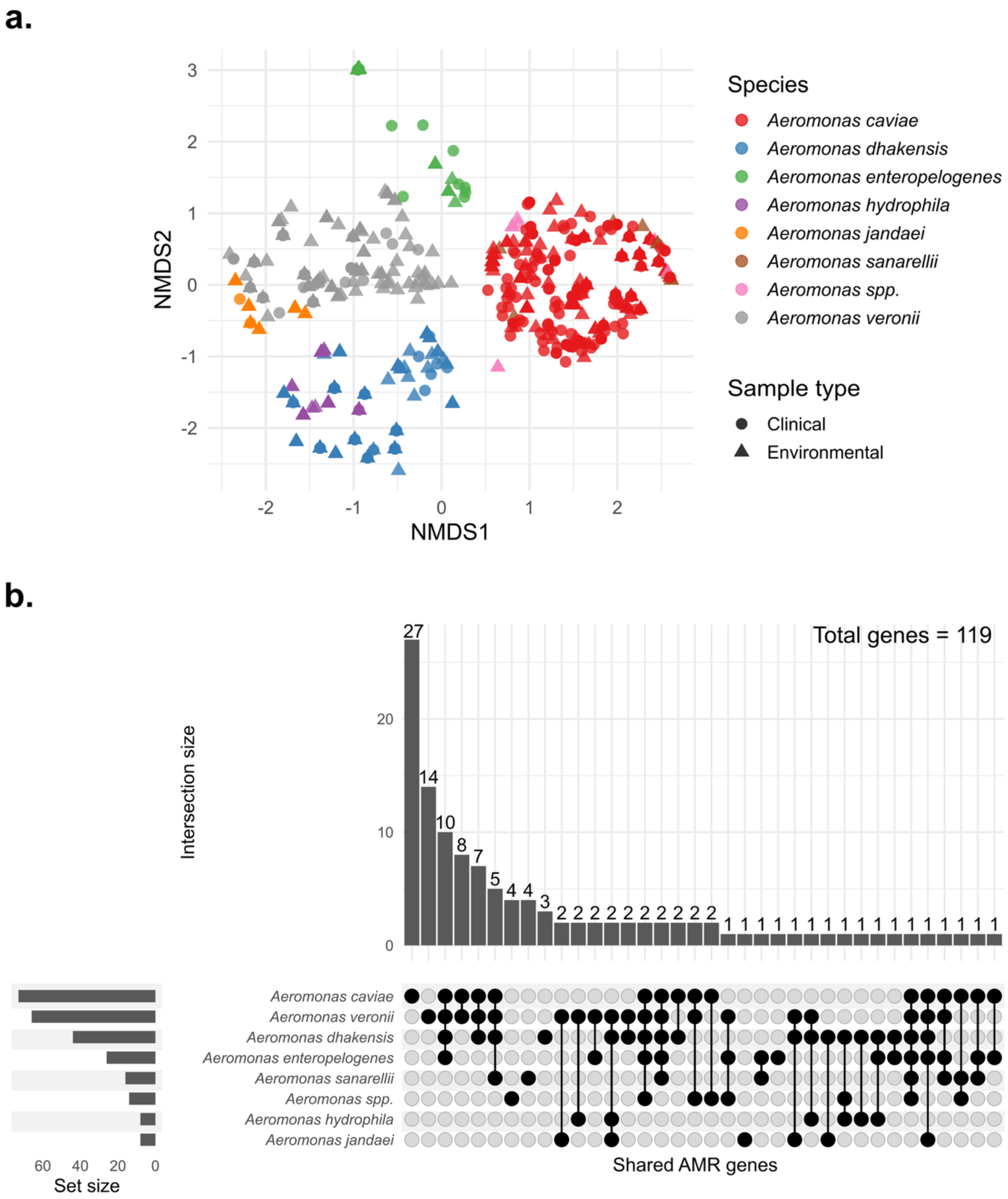

Supplementary Fig. 7: Distribution of antimicrobial resistance (AMR) genes across 1,438 *Aeromonas* species isolates from South Asia, including Bangladesh, India, and Pakistan. (a) Non-metric Multidimensional Scaling (NMDS) plot illustrating the variation in AMR gene profiles among *Aeromonas* species and sample types. Each point represents an isolate, coloured by species and shaped by sample type (clinical or environmental; see key). (b) UpSet plot showing the number of shared AMR genes across *Aeromonas* species.

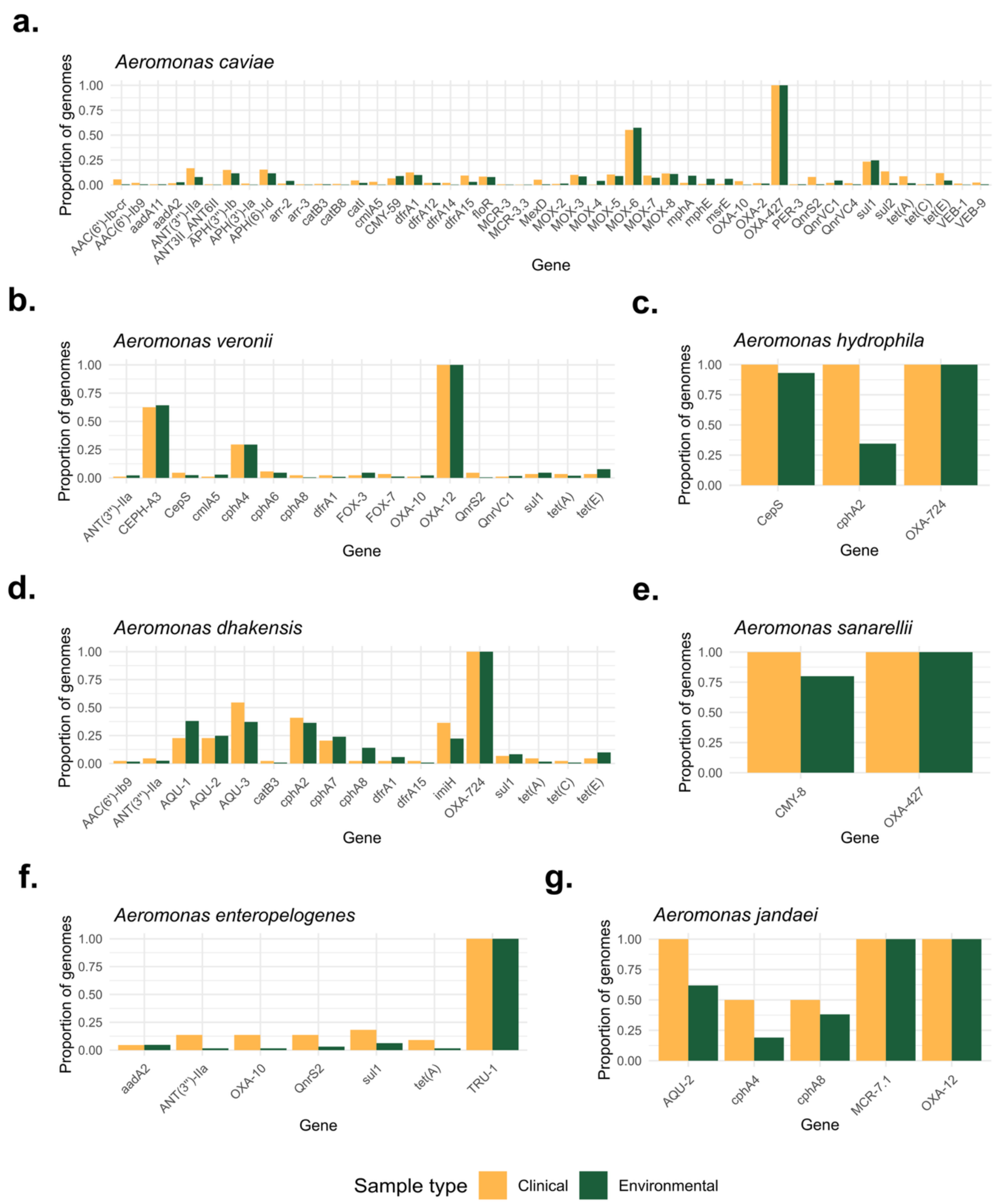

Supplementary Fig. 8: Distribution of shared antimicrobial resistance (AMR) genes among clinical and environmental *Aeromonas* isolates (*n* = 1,438) from South Asia, including Bangladesh, India, and Pakistan. Panels show data for (a) *A. caviae*, (b) *A. veronii*, (c) *A. hydrophila*, (d) *A. dhakensis*, (e) *A. sanarellii*, (f) *A. enteropelogenes*, and (g) *A. jandaei*, with bars coloured by sample type (see key).

Supplementary Table 1: Overview of 1,853 *Aeromonas* genomes included in this study.

Country	Genomes	Source (Count, Year of isolation)	Organisms (Count)	Study
Afghanistan	2	Stray dog (*Canis lupus familiaris*) faecal sample (2, 2015)	*A. caviae* (1), *A. hydrophila* (1)	Blackwell, G. A. *et al*., 2021[1]
Bangladesh	198	Drinking water (130, 2013), Human rectal swab (2, 2013), Human stool (1, 2013), Phytoplankton (1, 2005), Plankton (24, 2010–2015), Sediment (2, 2011), Water (37, 2004–2016), Zooplankton (1, 2006)	*A. caviae* (63), *A. dhakensis* (44), *A. enteropelogenes* (58), *A. jandaei* (3), *A. sanarellii* (1), *A. schubertii* (1), *A. veronii* (28)	This study
Brazil	1	Human stool (1, 2010)	*A. caviae* (1)	Blackwell, G. A. *et al*., 2021[1]
Canada	5	Fish sample (1, 2004), Wastewater (4, 2017)	*A. allosaccharophila* (1), *A. caviae* (1), *A. hydrophila* (1), *A. media* (1), *A. salmonicida* (1)	Blackwell, G. A. *et al*., 2021[1]
China	62	Fish sample (1, 2012), Sewage water (1, 2015), RAS Atlantic Salmon facility (60, 2012–2016)	*A. hydrophila* (2), *A. salmonicida* (60)	Blackwell, G. A. *et al*., 2021[1]
Denmark	100	Faeces (1, 2014), Unknown (99, 1980–2014)	*A. allosaccharophila* (1), *A. salmonicida* (99)	Blackwell, G. A. *et al*., 2021[1]
France	10	Aquarium tank (1, 2012), Human wound (3, 2012–2014), Lake water (1, Unknown), Wastewater (1, Unknown), *Hirudo verbana* crop (4, 2015)	*A. hydrophila* (2), *A. veronii* (8)	Blackwell, G. A. *et al*., 2021[1]
Germany	1	Unknown (1, Unknown)	*A. veronii* (1)	Blackwell, G. A. *et al*., 2021[1]
India	799	Drinking water (117, 2021–2023), Fish (1, 2015), Human stool (1, 2021), Lake water (26, 2020–2022), Pond water (47, 2020–2022), River water (599, 2020–2022), Water (8, 2023)	*A. caviae* (230), *A. dhakensis* (79), *A. enteropelogenes* (7), *A. hydrophila* (58), *A. jandaei* (18), *A. media* (4), *A. rivipollensis* (5), *A. sanarellii* (10), *A. taiwanensis* (2), *A. veronii* (386)	This study
Italy	1	Unknown (1, Unknown)	*A. veronii* (1)	Blackwell, G. A. *et al*., 2021[1]
Martinique	3	Human sample (3, Unknown)	*A. dhakensis* (3)	Blackwell, G. A. *et al*., 2021[1]
Mexico	1	Human urine (1, 2013)	*A. caviae* (1)	Blackwell, G. A. *et al*., 2021[1]
Pakistan	441	Human stool (441, 2008–2011)	*A. caviae* (284), *A. dhakensis* (43), *A. enteropelogenes* (21), *A. hydrophila* (1), *A. jandaei* (2), *A. sanarellii* (1), *A. taiwanensis* (1), *A. veronii* (88)	Klemm, E. J. *et al*., 2024[2]
Spain	1	Drinking water (1, Unknown)	*A. veronii* (1)	Blackwell, G. A. *et al*., 2021[1]
Switzerland	1	Thumb wound (1, 2016)	*A. salmonicida* (1)	Blackwell, G. A. *et al*., 2021[1]
Tanzania	2	Faeces (2, 2015)	*A. caviae* (2)	Blackwell, G. A. *et al*., 2021[1]
Thailand	1	Food (1, 2016)	*A. salmonicida* (1)	Blackwell, G. A. *et al*., 2021[1]
United Kingdom	2	Unknown (2, 1953 & 1991)	*A. salmonicida* (2)	Blackwell, G. A. *et al*., 2021[1]
United State	71	Channel catfish (1, 2004), Cow paddock straw (3, 2016) Medical instrument (1, Unknown), Fish sample (11, 1997 & 2006-2010), Human Peri-rectal (1, 2014), Human abdomen (2, 2015–2016), Human blood (1, 2015), Human wound (3, 2013 & Unknown), Human sample (7, 2014 & Unknown), Human stool (3, 2013), Human-assoc. habitat (2, 2013), Perirectal swab (1, 2013), Pond/Sediment (3, 2011), Waste water (9, 2014–2016), Zebrafish gut (2, 2008), Leech crop (20, 2002 & 2013-2014), Unknown (1, Unknown)	*A. allosaccharophila* (1), *A. bestiarum* (1), *A. caviae* (10), *A. hydrophila* (30), *A. salmonicida* (4), *A. veronii* (25)	Blackwell, G. A. *et al*., 2021[1]
Unknown	151	Catfish (8, 2010 & 2014–2015), Diseased channel catfish (5, 2013–2014), Duck (1, Unknown), Fish (Perch) spleen/head kidney (5, Unknown), Fish sample (6, Unknown), Rainbow trout sample (8, Unknown), River water (1, Unknown), Snail (1, Unknown), Zebrafish gut (2, Unknown), Leech gut (2, Unknown), Human Forehead abscess (1), Human Blood (4), Human Stool (1), Human Wound (1, Unknown), Unknown (105, Unknown)	*A. allosaccharophila* (2), *A. australiensis* (1), *A. bestiarum* (1), *A. bivalvium* (1), *A. caviae* (15), *A. dhakensis* (17), *A. diversa* (1), *A. encheleia* (1), *A. enteropelogenes* (19), *A. eucrenophila* (1), *A. fluvialis* (1), *A. hydrophila* (29), *A. jandaei* (2), *A. media* (2), *A. piscicola* (1), *A. popoffii* (1), *A. rivipollensis* (3), *A. rivuli* (1), *A. salmonicida* (8), *A. sanarellii* (4), *A. schubertii* (1), *A. simiae* (1), *A. sobria* (6), *Aeromonas sp.* (1), *A. sp900156095* (1), *A. taiwanensis* (1), *A. tecta* (1), *A. veronii* (28)	Blackwell, G. A. *et al*., 2021[1]

Supplementary Table 2: BAPS clusters and corresponding *Aeromonas* species.

BAPS clusters	Organism
Cluster-1	*Aeromonas schubertii, Aeromonas simiae, Aeromonas rivuli, Aeromonas diversa, Aeromonas bivalvium, Aeromonas sp900156095*
Cluster-2	*Aeromonas rivipollensis, Aeromonas media, Aeromonas tecta, Aeromonas eucrenophila, Aeromonas encheleia*
Cluster-3	*Aeromonas sanarellii, Aeromonas taiwanensis*
Cluster-4	*Aeromonas caviae*
Cluster-5	*Aeromonas hydrophila*
Cluster-6	*Aeromonas dhakensis*
Cluster-7	*Aeromonas popoffii, Aeromonas piscicola, Aeromonas bestiarum, Aeromonas sp.*
Cluster-8	*Aeromonas salmonicida*
Cluster-9	*Aeromonas enteropelogenes*
Cluster-10	*Aeromonas jandaei*
Cluster-11	*Aeromonas sobria*
Cluster-12	*Aeromonas fluvialis*
Cluster-13	*Aeromonas allosaccharophila*
Cluster-14	*Aeromonas australiensis*
Cluster-15	*Aeromonas veronii*
Cluster-16	*Aeromonas veronii*

Supplementary Table 3: Distribution of clinical and environmental *Aeromonas* isolates across sublineages (BAPS) from South Asia**,** including Bangladesh, India, and Pakistan.

Organism	BAPS_L2 (total *n*)	Clinical *n* (%)	Environmental *n* (%)
Aeromonas caviae (*n* = 577; Clinical = 286, Environmental = 291)	Cluster-4.1 (61)	32 (52.45%)	29 (47.54%)
	Cluster-4.2 (59)	27 (45.76%)	32 (54.24%)
	Cluster-4.3 (56)	15 (26.79%)	41 (73.21%)
	Cluster-4.4 (33)	17 (51.52%)	16 (48.48%)
	Cluster-4.5 (2)	1 (50.00%)	1 (50.00%)
	Cluster-4.6 (7)	3 (42.86%)	4 (57.14%)
	Cluster-4.7 (92)	41 (44.57%)	51 (55.43%)
	Cluster-4.8 (144)	105 (72.92%)	39 (27.08%)
	Cluster-4.9 (14)	4 (28.57%)	10 (71.43%)
	Cluster-4.10 (5)	0 (0.00%)	5 (100.00%)
	Cluster-4.11 (104)	41 (39.42%)	63 (60.58%)
Aeromonas veronii (*n* = 502; Clinical = 88, Environmental = 414)	Cluster-15.1 (2)	0 (0.00%)	2 (100.00%)
	Cluster-15.2 (4)	1 (25.00%)	3 (75.00%)
	Cluster-15.3 (20)	0 (0.00%)	20 (100.00%)
	Cluster-15.4 (3)	0 (0.00%)	3 (100.00%)
	Cluster-15.5 (4)	0 (0.00%)	4 (100.00%)
	Cluster-15.6 (14)	1 (7.14%)	13 (92.86%)
	Cluster-15.7 (1)	1 (100.00%)	0 (0.00%)
	Cluster-15.8 (1)	0 (0.00%)	1 (100.00%)
	Cluster-16.1 (453)	85 (18.77%)	368 (81.23%)
Aeromonas dhakensis (*n* = 166; Clinical = 44, Environmental = 121, Other = 1)	Cluster-6.1 (165)	44 (26.67%)	121 (73.33%)
Aeromonas enteropelogenes (*n* = 86; Clinical = 22, Environmental = 64)	Cluster-9.1 (78)	14 (17.95%)	64 (82.05%)
	Cluster-9.2 (8)	8 (100.00%)	0 (0.00%)
Aeromonas hydrophila (*n* = 59; Clinical = 1, Environmental = 58)	Cluster-5.1 (2)	0 (0.00%)	2 (100.00%)
	Cluster-5.2 (9)	0 (0.00%)	9 (100.00%)
	Cluster-5.3 (11)	0 (0.00%)	11 (100.00%)
	Cluster-5.4 (36)	1 (2.78%)	35 (97.22%)
	Cluster-5.5 (1)	0 (0.00%)	1 (100.00%)

Supplementary Table 4: Overview of Antimicrobial Resistance (AMR) genes identified in 1,853 *Aeromonas* species genomes.

Drug class/ Organism	*Aeromonas caviae*	*Aeromonas dhakensis*	*Aeromonas enteropelogenes*	*Aeromonas hydrophila*	*Aeromonas jandaei*	*Aeromonas salmonicida*	*Aeromonas sanarellii*	*Aeromonas spp.*	*Aeromonas veronii*
Aminoglycoside	*ANT(3'')-Iia, APH(6)-Id, APH(3'')-Ib, APH(3')-Ia, ANT(3'')-II-AAC(6')-Iid, aadA2, AAC(3)-Iie, AAC(6')-Ib10, AAC(6')-Ib9, AAC(6')-Ib7, aadA3, aadA15*	*AAC(6')-Ib9, ANT(3'')-Iia, aadA2*	*ANT(3'')-IIa*	*ANT(3'')-Iia, APH(6)-Id, APH(3'')-Ib, ANT(2'')-Ia, aadA2, APH(3')-Ia, AAC(3)-Iid, AAC(3)-IIIc, APH(3')-Iib, APH(3')-VI, AAC(6')-Iia, AAC(6')-Ib7*	_	*ANT(3'')-Iia, APH(6)-Id, APH(3'')-Ib ,aadA7, APH(3')-Ia, AAC(3)-Iie, aadA2, AAC(6')-Ib9, AAC(3)-Iid, AAC(6')-Ia, ANT(2'')-Ia*	*ANT(3'')-Iia, APH(6)-Id, APH(3'')-Ib*	*ANT(3'')-Iia, AAC(6')-Ib9, APH(3'')-Ib, aadA10, APH(6)-Id, ANT(2'')-Ia, AAC(6')-Iia*	*aadA2, APH(3')-Ia, AAC(6')-Ib7, AAC(6')-Iic, AAC(6')-Iaf, ANT(3'')-Iia, ANT(2'')-Ia, AAC(3)-Iid, APH(3'')-Ib, APH(6)-Id*
β-lactam (Carbapenem)	_	*cphA2, cphA7, imiH, cphA8*	_	*CEPH-A3, cphA6, imiS, cphA7, imiH, cphA2*	*CEPH-A3, cphA4, cphA8*	*cphA5*	_	*cphA8, cphA5, cphA6, cphA4, imiH, CEPH-A3*	*cphA4, CEPH-A3, cphA6, cphA8*
β-lactam (Cephalosporin)	*CTX-M-15*	*AQU-2, AQU-1, AQU-3*	_	*CepS_beta-lactamase, AQU-2*	*AQU-2, AQU-1*	*CTX-M-3*	_	*AQU-2*	*CepS_beta-lactamase*
β-lactam (Cephamycin)	*CMY-59*	_	_	_	_	*CMY-59*	*CMY-1, CMY-8*	_	_
β-lactam (Penam)	*CARB-3*	_	_	*CARB-3, CARB-12, FONA-5*	_	_	_	_	_
β-lactam (Cephalosporin and Cephamycin)	*FOX-5*	_	_	_	_	*FOX-2, FOX-4, FOX-3*	_	*FOX-2, FOX-7, FOX-8, FOX-1*	*FOX-3, FOX-2, FOX-7*
β-lactam (Cephalosporin and Penam)	*OXA-427, OXA-10, OXA-1, OXA-9*	*OXA-724, OXA-10*	*TRU-1, OXA-10*	*OXA-724, OXA-12, OXA-10*	*TRU-1, OXA-12*	*OXA-427, OXA-10, OXA-1*	*OXA-427, OXA-392*	*OXA-427, OXA-12, OXA-724, OXA-10, TRU-1, OXA-4, OXA-17*	*OXA-12, TRU-1, OXA-10, OXA-1*
β-lactam (Cephalosporin and Monobactam)	*VEB-9*	_	_	_	_	*VEB-1*	_	_	*VEB-1*
β-lactam (Cephalosporin, Penam and Penem)	_	_	_	_	_	*SCO-1*	_	_	_
β-lactam (Carbapenem, Cephalosporin and Penam)	*OXA-2, GES-14*	_	_	*SHV-134, GES-14*	_	_	_	*GES-1, GES-14*	*SHV-134*
β-lactam (Cephalosporin, Cephamycin and Penam)	*MOX-6, MOX-5, MOX-8, MOX-2, MOX-7, MOX-4, MOX-3*	_	_	_	_	_	_	*MOX-9, MOX-6, MOX-2*	_
β-lactam (Carbapenem, Cephalosporin, Monobactam and Penam)	*KPC-1*	_	_	*KPC-1*	_	_	_	_	_
β-lactam (Cephalosporin, Monobactam, Penam and Penem)	*TEM-150*	_	*TEM-1*	*TEM-26*	_	*TEM-1*	_	_	*TEM-1, TEM-116, TEM-150*
β-lactam (Carbapenem, Cephalosporin, Monobactam, Penam and Penem)	*PER-3*	_	_	_	_	_	_	_	_

Diaminopyrimidine	*dfrA15, dfrA12, dfrA1, dfrA23*	*dfrA14, dfrA15, dfrA12*	*dfrA10, dfrA7, dfrA15*	*dfrA15, dfrA19, dfrA12, dfrA1*	–	*dfrA1, dfrA14, dfrA12, dfrA5*	*dfrB5*	–	*dfrA1, dfrA12, dfrA17, dfrA14*
Fluoroquinolone	*QnrS2, QnrVC4, QnrA1*	*QnrVC4*	*qacH, QnrVC4, QnrS2*	*QnrB5, QnrS2, QnrVC6*	–	*QnrVC4, QnrVC6, qacH*	–	*qacH*	*QnrB4, QnrS2, QepA2*
Macrolide	*mphA, mphE, mphF*	*mphA*	–	*mphE, mphA*	–	*mphE, mphA*	*EreA2*	*mphE*	*mphA, EreA2*
Nucleoside	–	–	–	–	–	–	–	–	–
Peptide	*MCR-3, MCR-3.12, MCR-3.3*	–	–	*MCR-5*	*MCR-7.1*	*MCR-3, MCR-3.12*	–	*MCR-3.6, MCR-3, MCR-3.8, MCR-7.1, MCR-3.12*	*MCR-3, MCR-3.12, MCR-3.6, MCR-3.3*
Phenicol	*floR, cmlB1, cmlA1, catB3, Vibrio_anguillarum_chloramphenicol_acetyltransferas1-651/651, catB8, catI, cmlA5, cmlA6, catB11, catII*	*cmlA5*	*cmlA5*	*cmlA5, catB8, catB3, catB11, cmlA6, floR*	–	*floR, Ecol_catII, catB3, cmlA5*	*catB3, floR*	*cmlA5, catB3*	*catII, floR, cmx, cmlA5, catB3*
Rifamycin	*arr-3, arr-2*	–	–	*arr-3*	–	*arr-3*	*arr-2*	–	*arr-2, arr-3*
Sulphonamide	*sul2, sul1*	*sul1*	*sul1*	*sul1, sul2*	–	*sul1, sul2*	*sul1, sul2*	*sul1*	*sul1, sul2*
Tetracycline	*tet(E), tet(A), tet(C), tet(G), tet(31)*	*tet(A), tet(E), tet(C)*	*tet(E), tet(A)*	*tet(A), tet(E), tet(C)*	*tet(A)*	*tet(A), tet (E)*	–	*tet(A), tet (E)*	*tet(E), tet(A), tet(D)*
Aminoglycoside, Fluoroquinolone	*AAC(6')-Ib-cr*		–	*AAC(6')-Ib-cr*	–	–	–	–	*AAC(6')-Ib-cr*
Aminoglycoside, β-lactam (Cephalosporin), Macrolide, Peptide, Rifamycin, Tetracycline	–	–	*Kpne_KpnE*	–	–	–	–	–	–
Fluoroquinolone, Macrolide and β-lactam (Penam)	–	–	–	–	–	–	–	*CRP*	–
Lincosamide, Macrolide, Oxazolidinone, Phenicol, Pleuromutilin, Streptogramin, Tetracycline	*msrE*	–	–	*msrE*	–	*msrE*	–	*msrE*	–
Aminocoumarin, Aminoglycoside, β-lactam (Cephalosporin, Penem), Diaminopyrimidine, Fluoroquinolone, Macrolide, Phenicol and Tetracycline	*MexD*	–	–	*MexD*	–	*MexD*	–	–	–

Supplementary Table 5: Description of the identified β-lactamase genes in 1,853 *Aeromonas* species genomes.

Ambler class	Genes	*A. caviae* (*n* = 608)	*A. dhakensis* (*n* = 186)	*A. enteropelogenes* (*n* = 105)	*A. hydrophila* (*n* = 124)	*A. jandaei* (*n* = 25)	*A. salmonicida* (*n* = 176)	*A. sanarellii* (*n* = 16)	*Aeromonas* spp. (*n* = 47)	*A. veronii* (*n* = 566)
Class A	*CARB-12*	0	0	0	**1**	0	0	0	0	0
	CARB-3	**1**	0	0	**1**	0	0	0	0	0
	CTX-M-15	**1**	0	0	0	0	0	0	0	0
	CTX-M-3	0	0	0	0	0	**1**	0	**4**	0
	FONA-5	0	0	0	**1**	0	0	0	0	0
	GES-1	0	0	0	0	0	0	0	**1**	0
	GES-14	**1**	0	0	**1**	0	0	0	**1**	0
	KPC-1	**6**	0	0	**9**	0	0	0	0	0
	PER-3	**4**	0	0	0	0	0	0	0	**5**
	RSA-1	**11**	**7**	0	0	0	0	0	0	**2**
	SCO-1	0	0	0	0	0	**1**	0	0	0
	SHV-134	0	0	0	**1**	0	0	0	0	**1**
	TEM-1	0	0	**1**	0	0	**1**	0	**4**	**4**
	TEM-116	0	0	0	0	0	0	0	0	**1**
	TEM-150	**1**	0	0	0	0	0	0	0	**1**
	TEM-26	0	0	0	**3**	0	0	0	0	0
	VEB-1	**5**	0	0	0	0	**1**	0	0	**13**
	VEB-2	0	0	0	0	0	0	0	0	**1**
	VEB-9	**9**	0	0	0	0	0	0	0	**4**
Class B	*CEPH-A3*	0	**4**	0	**30**	**10**	0	0	**4**	**362**
	cphA2	0	**72**	0	**31**	0	0	0	0	0
	cphA4	0	0	0	0	**5**	0	0	**5**	**167**
	cphA5	0	0	0	0	0	**113**	0	**2**	0
	cphA6	0	0	0	**2**	0	0	0	**1**	**28**
	cphA7	0	**41**	**1**	**25**	0	0	0	0	0
	cphA8	0	**19**	0	0	10	0	0	**3**	**4**
	imiH	0	**50**	0	**32**	0	0	0	**2**	**1**
	imiS	0	0	0	**2**	0	0	0	0	0
	VIM-6	**1**	0	0	0	0	0	0	0	0
Class C	*AQU-1*	0	**62**	0	0	**4**	0	0	0	0
	AQU-2	0	**49**	0	**24**	**16**	0	0	0	**1**
	AQU-3	0	**75**	0	0	0	0	0	0	**1**
	CepS	0	0	0	**100**	0	0	0	0	**14**
	CMY-1	0	0	0	0	0	0	**6**	0	0
	CMY-2	0	0	0	0	0	0	0	0	**1**
	CMY-59	**46**	0	**2**	0	0	**1**	**1**	0	**4**
	CMY-8	0	0	0	0	0	0	**10**	0	0
	DHA-1	**1**	0	0	0	0	0	0	0	**1**
	FOX-1	0	0	0	0	0	0	0	**1**	**1**
	FOX-2	0	0	0	0	0	**168**	0	**8**	**1**
	FOX-3	0	0	0	0	0	**3**	0	0	**21**
	FOX-4	0	0	0	0	0	**5**	0	0	0
	FOX-5	**1**	0	0	0	0	0	0	0	0
	FOX-7	0	0	0	0	0	0	0	**2**	**9**
	FOX-8	0	0	0	0	0	0	0	**1**	0
	MOX-2	**7**	0	0	0	0	0	0	**1**	0
	MOX-3	**55**	0	0	0	0	0	0	0	0
	MOX-4	**13**	0	0	0	0	0	0	0	0
	MOX-5	**60**	0	0	0	0	0	0	0	0
	MOX-6	**342**	0	0	0	0	0	0	**4**	**4**
	MOX-7	**54**	0	0	0	0	0	0	0	0
	MOX-8	**68**	0	0	0	0	0	0	0	0
	MOX-9	0	0	0	0	0	0	0	**15**	0
	TRU-1	0	0	**105**	0	**1**	0	0	0	**5**
Class D	*OXA-1*	**1**	0	0	0	0	**27**	0	0	**1**
	OXA-10	**12**	**2**	**8**	**1**	0	**25**	0	**1**	**11**
	OXA-181	**5**	0	0	0	0	0	0	0	0
	OXA-12	0	0	0	**2**	**25**	0	0	**13**	**565**
	OXA-17	0	0	0	0	0	0	0	**1**	0
	OXA-2	**14**	0	0	0	0	0	0	0	0
	OXA-392	0	0	0	0	0	0	**1**	0	0
	OXA-21	0	0	0	0	0	0	0	0	**1**
	OXA-4	0	0	0	0	0	0	0	**1**	0
	OXA-427	**608**	0	0	0	0	**176**	**16**	**26**	0
	OXA-724	0	**186**	0	**122**	0	0	0	**4**	0
	OXA-9	**2**	0	0	0	0	0	0	0	0

Supplementary Table 6: Description of the unique and shared antimicrobial resistance (AMR) genes across 1,438 *Aeromonas* species isolates from South Asia, including Bangladesh, India, and Pakistan.

Organism	No. of Gene	AMR Genes
A. caviae	27	*AAC(3)-IIe, CTX-M-15, MCR-3.10, MCR-3.3, MOX-2, MOX-3, MOX-4, MOX-5, MOX-7, MOX-8, MexD, OXA-1, OXA-181, OXA-2, OXA-9, SAT-1, TEM-150, VIM-6, Vang_ACT_CHL, aadA11, cmlA4, cmlB1, dfrA23, dfrB1, floR, tet(31), tet(G)*
A. dhakensis	3	*AAC(6')-Ib8, mgrA, tet(K)*
A. enteropelogenes	1	*Kpne_KpnE*
A. jandaei	1	*MCR-7.1*
A. sanarellii	4	*CMY-1, CMY-8, OXA-392, dfrB5*
A. spp.	4	*CTX-M-3, MOX-9, QnrVC6, dfrA27*
A. veronii	14	*AAC(6')-IIa, AAC(6')-Ib4, CMY-2, FOX-1, FOX-2, FOX-3, FOX-7, MCR-3.12, MCR-3.8, OXA-21, VEB-2, aadA5, dfrA17, dfrB3*
A. caviae, A. dhakensis	2	*dfrB4, sul2*
A. caviae, A. enteropelogenes	1	*cmlA1*
A. caviae, A. spp.	2	*AAC(3)-IId, arr-3*
A. caviae, A. veronii	8	*ANT3II_ANT6II, APH(3')-Ia, DHA-1, MCR-3, MCR-3.6, PER-3, VEB-1, VEB-9*
A. dhakensis, A. enteropelogenes	1	*dfrA5*
A. dhakensis, A. hydrophila	1	*cphA2*
A. dhakensis, A. jandaei	1	*AQU-1*
A. dhakensis, A. veronii	2	*AQU-3, aadA16*
A. enteropelogenes, A. sanarellii	1	*EreA2*
A. enteropelogenes, A. veronii	2	*TRU-1, dfrA10*
A. hydrophila, A. veronii	2	*CepS, cphA6*
A. jandaei, A. veronii	2	*OXA-12, cphA4*
A. caviae, A. dhakensis, A. veronii	7	*AAC(6')-Ib9, ANT(2'')-Ia, RSA-1, catB8, dfrA14, mphE, msrE*
A. caviae, A. enteropelogenes, A. sanarellii	1	*catI*
A. caviae, A. sanarellii, A. spp.	1	*OXA-427*
A. caviae, A. spp., A. veronii	2	*AAC(6')-Ib-cr, MOX-6*
A. dhakensis, A. enteropelogenes, A. hydrophila	1	*cphA7*
A. dhakensis, A. hydrophila, A. spp.	1	*OXA-724*
A. dhakensis, A. hydrophila, A. veronii	1	*imiH*
A. dhakensis, A. jandaei, A. veronii	1	*cphA8*
A. enteropelogenes, A. spp., A. veronii	1	*TEM-1*
A. caviae, A. dhakensis, A. enteropelogenes, A. veronii	10	*OXA-10, QnrVC1, QnrVC4, aadA2, catII, cmlA5, dfrA12, dfrA15, qacH, tet(C)*
A. caviae, A. dhakensis, A. sanarellii, A. veronii	5	*APH(3'')-Ib, APH(6)-Id, arr-2, catB3, dfrA1*
A. caviae, A. enteropelogenes, A. sanarellii, A. veronii	1	*CMY-59*
A. dhakensis, A. hydrophila, A. jandaei, A. veronii	2	*AQU-2, CEPH-A3*
A. caviae, A. dhakensis, A. enteropelogenes, A. jandaei, A. veronii	1	*tet(A)*
A. caviae, A. dhakensis, A. enteropelogenes, A. sanarellii, A. veronii	2	*ANT(3'')-IIa, QnrS2*
A. caviae, A. dhakensis, A. enteropelogenes, A. spp., A. veronii	2	*mphA, tet(E)*
A. caviae, A. dhakensis, A. enteropelogenes, A. sanarellii, A. spp., A. veronii	1	*sul1*

Supplementary Table 7: Description of the unique and shared antimicrobial resistance (AMR) genes among clinical and environmental *Aeromonas* isolates (*n* = 1,438) from South Asia, including Bangladesh, India, and Pakistan.

Organism	*A. caviae*	*A. dhakensis*	*A. enteropelogenes*	*A. hydrophila*	*A. jandaei*	*A. sanarellii*	*Aeromonas spp.*	*A. veronii*
Genes in clinical isolates	18	5	11	0	1	0	0	4
	catII, qacH, MCR-3.6, DHA-1, AAC(3)-IId, tet(31), AAC(3)-IIe, CTX-M-15, OXA-1, OXA-9, TEM-150, Vang_ACT_CHL, SAT-1, tet(G), dfrA23, MCR-3.10, dfrB4, VIM-6	*catII, QnrS2, ANT(2'')-Ia, catB8, dfrB4*	*cphA7, CMY-59, dfrA15, cmlA5, TEM-1, cmlA1, tet(C), EreA2, QnrVC1, catI, dfrA5*		*tet(A)*			*AQU-3, FOX-2, dfrA17, CMY-2*
Genes in environmental isolates	7	22	8	5	2	14	12	45
	cmlB1, cmlA1, RSA-1, ANT(2'')-Ia, OXA-181, dfrB1, cmlA4	*mphA, CEPH-A3, sul2, APH(3'')-Ib, APH(6)-Id, dfrA12, OXA-10, QnrVC4, cmlA5, dfrA14, qacH, aadA2, arr-2, QnrVC1, RSA-1, mphE, msrE, mgrA, tet(K), AAC(6')-Ib8, aadA16, dfrA5*	*tet(E), mphA, catII, dfrA12, QnrVC4, Kpne_KpnE, dfrA10, qacH*	*AQU-2, cphA7, CEPH-A3, imiH, cphA6*	*AQU-1, CEPH-A3*	*CMY-59, sul1, ANT(3'')-IIa, APH(3'')-Ib, APH(6)-Id, QnrS2, dfrA1, EreA2, OXA-392, arr-2, catB3, dfrB5, CMY-1, catI*	*OXA-724, tet(E), mphA, sul1, AAC(6')-Ib-cr, TEM-1, arr-3, MOX-9, CTX-M-3, QnrVC6, dfrA27, AAC(3)-IId*	*AQU-2, MOX-6, mphA, CMY-59, TRU-1, catII, dfrA15, AAC(6')-Ib-cr, APH(3'')-Ib, APH(3')-Ia, APH(6)-Id, dfrA12, imiH, AAC(6')-Ib9, QnrVC4, dfrA14, TEM-1, dfrA10, qacH, MCR-3, MCR-3.12, MCR-3.6, aadA2, tet(C), arr-2, catB3, ANT3II_ANT6II, PER-3, RSA-1, mphE, msrE, VEB-9, ANT(2'')-Ia, VEB-1, aadA16, aadA5, catB8, AAC(6')-Ib4, FOX-1, VEB-2, DHA-1, MCR-3.8, AAC(6')-IIa, OXA-21, dfrB3*
Genes shared between clinical and environmental isolates	48	17	7	3	5	2	2	17
	tet(A), tet(E), MOX-6, OXA-427, mphA, CMY-59, MOX-5, MOX-8, MOX-2, sul2, dfrA15, sul1, ANT(3'')-IIa, MOX-7, AAC(6')-Ib-cr, APH(3'')-Ib, APH(3')-Ia, APH(6)-Id, MexD, QnrS2, dfrA12, floR, MOX-4, AAC(6')-Ib9, OXA-10, QnrVC4, cmlA5, dfrA14, MCR-3, dfrA1, MOX-3, aadA2, tet(C), arr-2, catB3, ANT3II_ANT6II, PER-3, arr-3, QnrVC1, mphE, msrE, VEB-9, catI, OXA-2, aadA11, VEB-1, catB8, MCR-3.3	*AQU-2, OXA-724, cphA2, tet(A), AQU-1, cphA7, tet(E), AQU-3, dfrA15, sul1, ANT(3'')-IIa, imiH, AAC(6')-Ib9, cphA8, dfrA1, tet(C), catB3*	*tet(A), TRU-1, sul1, ANT(3'')-IIa, QnrS2, OXA-10, aadA2*	*OXA-724, cphA2, CepS*	*AQU-2, OXA-12, cphA4, cphA8, MCR-7.1*	*OXA-427, CMY-8*	*MOX-6, OXA-427*	*tet(A), tet(E), OXA-12, cphA4, CEPH-A3, sul1, ANT(3'')-IIa, QnrS2, OXA-10, cmlA5, FOX-3, cphA8, dfrA1, CepS, cphA6, QnrVC1, FOX-7*

Supplementary Table 8: Description of the primer sequence used for *in silico* PCR.

Virulence gene	Primer sequence (5′→3′)	Reference
ast	F: ATGCACGCACGTACCGCC<br>R: ATATCCGGTCG TCG CTCTTG GT	Nhinh et al., 2021[3]
lip	F: ATCTTCTCCGACTGGTTCGG<br>R: CCGTGCCAGGACTGGGTCTT	Kingombe et al., 1999[4]
ela	F: ACACGGTCAAGGAGATCAAC<br>R: CGCTGGTGTTGGCCAGCAGG	Kingombe et al., 1999[4]
act	F: GAGAAGGTGACCACCAAGAACA<br>R: AACTGACATCGGCCTTGAACTC	Kingombe et al., 1999[4]
alt	F: GCACGGCGTGACTTCGGTGA<br>R: ACCGCGGTCTTGCAGTTGGG	Kingombe et al., 1999[4]
aerA	F: AACCGAACTCTCCAT<br>R: CGCCTTGTCCTTGTA	Nhinh et al., 2021[3]
hlyA	F: GGCCGGTGGCCCGAAGATACGGG<br>R: GGCGGCGCCGGACGAGACGGG	Nhinh et al., 2021[3]
fla	F: TCCAACCGTYTGACCTC<br>R: GMYTGGTTGCGRATGGT	Nhinh et al., 2021[3]
laf	F: GGTCTGCGCATCCAACTC<br>R: GCTCCAGACGGTTGATG	Kingombe et al., 1999[4]
ascF-G	F: ATGAGGTCATCTGCTCGCGC<br>R: GGAGCACAACCATGGCTG AT	Kingombe et al., 1999[4]

Supplementary References:

1. Blackwell, G. A. *et al.* Exploring bacterial diversity via a curated and searchable snapshot of archived DNA sequences. *PLoS Biol* **19**, (2021).
2. Klemm, E. J. *et al.* Genomic analysis of clinical Aeromonas isolates reveals genetic diversity but little evidence of genetic determinants for diarrhoeal disease. *Microb Genom* **10**, 1211 (2024).
3. Nhinh, D. T. *et al.* Prevalence, Virulence Gene Distribution and Alarming the Multidrug Resistance of Aeromonas hydrophila Associated with Disease Outbreaks in Freshwater Aquaculture. *Antibiotics* 10, 532 (2021).
4. Kingombe, C. I. Bin *et al.* PCR detection, characterization, and distribution of virulence genes in Aeromonas spp. *Appl Environ Microbiol* 65, (1999).
