# Supplementary material for: Aeromonas in South Asia: genomic insights into an environmental pathogen and reservoir of antimicrobial resistance
Source: Nat Commun. 2026 Jan 31;17:2214. doi: 10.1038/s41467-026-68712-w (PMC12963386; doi:10.1038/s41467-026-68712-w)
Supplement: Supplementary file 2 — Description of Additional Supplementary Files [file 41467_2026_68712_MOESM2_ESM.pdf]

## **Description of Additional Supplementary Files**

| | |
|---|---|
| **Supplementary Data 1** | Detailed metadata and integrated analysis outputs for 1,853 *Aeromonas* genomes |
| **Supplementary Data 2** | Genome taxonomy assignments using GTDB-Tk for 1,853 *Aeromonas* genomes |
| **Supplementary Data 3** | Genome taxonomy assignment for representative *Aeromonas* genomes using both digital DNA–DNA hybridization (dDDH) and GTDB-Tk |
