# Supplementary material for: Aeromonas in South Asia: genomic insights into an environmental pathogen and reservoir of antimicrobial resistance
Source: Nat Commun. 2026 Jan 31;17:2214. doi: 10.1038/s41467-026-68712-w (PMC12963386; doi:10.1038/s41467-026-68712-w)
Supplement: Supplementary file 6 — Reporting Summary [file 41467_2026_68712_MOESM6_ESM.pdf]

nature portfolio

Corresponding author(s): Nicholas R. Thomson

Last updated by author(s): Dec 15, 2025

Reporting Summary

Nature Portfolio wishes to improve the reproducibility of the work that we publish. This form provides structure for consistency and transparency in reporting. For further information on Nature Portfolio policies, see our Editorial Policies and the Editorial Policy Checklist.

Statistics

For all statistical analyses, confirm that the following items are present in the figure legend, table legend, main text, or Methods section.

n/a	Confirmed	
☐	☒	The exact sample size (*n*) for each experimental group/condition, given as a discrete number and unit of measurement
☐	☒	A statement on whether measurements were taken from distinct samples or whether the same sample was measured repeatedly
☒	☐	The statistical test(s) used AND whether they are one- or two-sided<br>*Only common tests should be described solely by name; describe more complex techniques in the Methods section.*
☐	☒	A description of all covariates tested
☒	☐	A description of any assumptions or corrections, such as tests of normality and adjustment for multiple comparisons
☐	☒	A full description of the statistical parameters including central tendency (e.g. means) or other basic estimates (e.g. regression coefficient) AND variation (e.g. standard deviation) or associated estimates of uncertainty (e.g. confidence intervals)
☒	☐	For null hypothesis testing, the test statistic (e.g. *F*, *t*, *r*) with confidence intervals, effect sizes, degrees of freedom and *P* value noted<br>*Give P values as exact values whenever suitable.*
☐	☒	For Bayesian analysis, information on the choice of priors and Markov chain Monte Carlo settings
☒	☐	For hierarchical and complex designs, identification of the appropriate level for tests and full reporting of outcomes
☒	☐	Estimates of effect sizes (e.g. Cohen's *d*, Pearson's *r*), indicating how they were calculated

Our web collection on statistics for biologists contains articles on many of the points above.

Software and code

Policy information about availability of computer code

Data collection	No software was used for data collection.
Data analysis	The following software, databases, and R packages were used to analyze the data presented in this study:<br>Quality control: FastQC (v0.11.4), MultiQC (v1.17), Quast (v5.0.2), CheckM (v1.1.2)<br>Genome assembly and annotation: SPAdes (v3.9.0), PROKKA (v1.4.5)<br>Taxonomic classification and genome-relatedness indices: Kraken2 (v2.2), Bracken (v2.6.2) using Standard RefSeq database (archaea, bacteria, viruses, plasmids, human); GTDB-Tk (v2.4.1) with Genome Taxonomy Database R226; FastANI (v1.3)<br>AMR and virulence gene screening: ABRicate (v1.0.1) with CARD (v3.2.4, August 2022); in_silico_pcr (v1.0.0)<br>Sequence typing: MLST tool (v2.9) with Aeromonas MLST scheme<br>Pangenome analysis and population structure: panaroo (v1.3.0); fastBAPS (v1.0.6)<br>Phylogenetic analysis and metadata annotation: SNP-sites (v2.51), IQ-TREE (v2.1); iTOL (v5.6)<br>Data visualization, dimensionality reduction (PCA, NMDS), hierarchical clustering, and data manipulation were performed in R (v4.4.3) using the following packages: ComplexHeatmap; stats (prcomp); vegan; ggplot2; dplyr; tidyr; scales; ComplexUpset; forcats; RColorBrewer; stringr; svglite.

For manuscripts utilizing custom algorithms or software that are central to the research but not yet described in published literature, software must be made available to editors and reviewers. We strongly encourage code deposition in a community repository (e.g. GitHub). See the Nature Portfolio guidelines for submitting code & software for further information.

Data

Policy information about availability of data

All manuscripts must include a data availability statement. This statement should provide the following information, where applicable:

- Accession codes, unique identifiers, or web links for publicly available datasets
- A description of any restrictions on data availability
- For clinical datasets or third party data, please ensure that the statement adheres to our policy

The read data generated in this study have been deposited in the EMBL Nucleotide Sequence Database (European Nucleotide Archive, http://www.ebi.ac.uk/ena). BioSample and sequence accession numbers along with related metadata for all strains sequenced in this study, as well as strains obtained from public repositories (NCBI/ENA databases), are listed in Supplementary Data- 1.

Research involving human participants, their data, or biological material

Policy information about studies with human participants or human data. See also policy information about sex, gender (identity/presentation), and sexual orientation and race, ethnicity and racism.

Reporting on sex and gender	*Use the terms sex (biological attribute) and gender (shaped by social and cultural circumstances) carefully in order to avoid confusing both terms. Indicate if findings apply to only one sex or gender; describe whether sex and gender were considered in study design; whether sex and/or gender was determined based on self-reporting or assigned and methods used. Provide in the source data disaggregated sex and gender data, where this information has been collected, and if consent has been obtained for sharing of individual-level data; provide overall numbers in this Reporting Summary. Please state if this information has not been collected. Report sex- and gender-based analyses where performed, justify reasons for lack of sex- and gender-based analysis.*
Reporting on race, ethnicity, or other socially relevant groupings	*Please specify the socially constructed or socially relevant categorization variable(s) used in your manuscript and explain why they were used. Please note that such variables should not be used as proxies for other socially constructed/relevant variables (for example, race or ethnicity should not be used as a proxy for socioeconomic status). Provide clear definitions of the relevant terms used, how they were provided (by the participants/respondents, the researchers, or third parties), and the method(s) used to classify people into the different categories (e.g. self-report, census or administrative data, social media data, etc.) Please provide details about how you controlled for confounding variables in your analyses.*
Population characteristics	*Describe the covariate-relevant population characteristics of the human research participants (e.g. age, genotypic information, past and current diagnosis and treatment categories). If you filled out the behavioural & social sciences study design questions and have nothing to add here, write "See above."*
Recruitment	*Describe how participants were recruited. Outline any potential self-selection bias or other biases that may be present and how these are likely to impact results.*
Ethics oversight	*Identify the organization(s) that approved the study protocol.*

Note that full information on the approval of the study protocol must also be provided in the manuscript.

Field-specific reporting

Please select the one below that is the best fit for your research. If you are not sure, read the appropriate sections before making your selection.

☒ Life sciences ☐ Behavioural & social sciences ☐ Ecological, evolutionary & environmental sciences

For a reference copy of the document with all sections, see nature.com/documents/nr-reporting-summary-flat.pdf

Life sciences study design

All studies must disclose on these points even when the disclosure is negative.

Sample size	No formal sample size calculation was performed, as this study is based on the analysis of available Aeromonas genome sequences rather than a controlled experimental design. We included a total of 1,853 genomes, comprising 996 genomes sequenced in this study from cholera-endemic regions of Bangladesh (2004–2016) and Northern India (2020–2023) from diverse environmental sources, and 857 genomes obtained from previously published studies deposited in the European Nucleotide Archive and GenBank (Methods, Supplementary Table 1, Supplementary Data 1).<br><br>The sample size reflects all genomes that met quality requirements and had sufficient metadata. We sequenced the largest collection of environmental Aeromonas genomes from India and Bangladesh to capture environmental diversity. To enable comparison of clinical and environmental isolates in South Asia, we included the largest available dataset of clinical genomes from Pakistan, derived from children with moderate to severe diarrhoea and matched healthy controls. Publicly available genomes from other regions were also incorporated to provide a global context and capture species-level diversity within the Aeromonas genus. This dataset allowed comparative genomic analyses across regions, sources, and Aeromonas species.

Data exclusions	We excluded 5% of genomes due to failing quality control.
Replication	This study is based on an integrated analysis of newly generated genomes alongside publicly available genome data. All genome data are deposited in public repositories, and detailed information, including accession numbers and associated metadata, is provided in Supplementary Data-1. Details on the software used for bioinformatics analyses are described in the Methods. Additionally, all pipelines, parameters, and scripts (command-line and R code), together with metadata and genome analysis outputs, have been deposited on Figshare (https://doi.org/10.6084/m9.figshare.30833858), enabling full reproducibility of the analyses.<br><br>The results can be independently reproduced using the provided data and scripts.
Randomization	Randomization was not applicable to this study, as this is an observational study based on genome data from newly generated and previously published isolates.
Blinding	Sequencing teams were blind to sample metadata (source, country, date).

Reporting for specific materials, systems and methods

We require information from authors about some types of materials, experimental systems and methods used in many studies. Here, indicate whether each material, system or method listed is relevant to your study. If you are not sure if a list item applies to your research, read the appropriate section before selecting a response.

Materials & experimental systems

n/a	Involved in the study
☒	☐ Antibodies
☒	☐ Eukaryotic cell lines
☒	☐ Palaeontology and archaeology
☒	☐ Animals and other organisms
☒	☐ Clinical data
☒	☐ Dual use research of concern
☒	☐ Plants

Methods

n/a	Involved in the study
☒	☐ ChIP-seq
☒	☐ Flow cytometry
☒	☐ MRI-based neuroimaging

Plants

Seed stocks	*Report on the source of all seed stocks or other plant material used. If applicable, state the seed stock centre and catalogue number. If plant specimens were collected from the field, describe the collection location, date and sampling procedures.*
Novel plant genotypes	*Describe the methods by which all novel plant genotypes were produced. This includes those generated by transgenic approaches, gene editing, chemical/radiation-based mutagenesis and hybridization. For transgenic lines, describe the transformation method, the number of independent lines analyzed and the generation upon which experiments were performed. For gene-edited lines, describe the editor used, the endogenous sequence targeted for editing, the targeting guide RNA sequence (if applicable) and how the editor was applied.*
Authentication	*Describe any authentication procedures for each seed stock used or novel genotype generated. Describe any experiments used to assess the effect of a mutation and, where applicable, how potential secondary effects (e.g. second site T-DNA insertions, mosiacism, off-target gene editing) were examined.*
